# Prevalence of malaria in an area receiving seasonal malaria chemoprevention in Niger

**DOI:** 10.1186/s12936-021-03953-2

**Published:** 2021-10-24

**Authors:** Matthew E. Coldiron, Bachir Assao, Ousmane Guindo, Nathan Sayinzoga-Makombe, Alena Koscalova, Esther Sterk, Michel Quere, Iza Ciglenecki, Ann Mumina, Salifou Atti, Céline Langendorf, Rebecca F. Grais

**Affiliations:** 1 Epicentre, 14-34 Avenue Jean Jaurès, Paris, France; 2 Epicentre, Maradi, Niger; 3 Médecins Sans Frontières, 70 rue de Lausanne, Geneva, Switzerland; 4 Médecins Sans Frontières, Niamey, Niger; 5 Ministry of Public Health, Magaria, Niger

**Keywords:** Malaria, Seasonal malaria chemoprevention, Niger

## Abstract

**Background:**

Malaria transmission is highly seasonal in Niger. Despite the introduction of seasonal malaria chemoprevention (SMC) in the Magaria District, malaria incidence remains high, and the epidemiology of malaria in the community is not well-understood.

**Methods:**

Four cross-sectional, household-based malaria prevalence surveys were performed in the Magaria District of Niger between October 2016 and February 2018. Two occurred during the peak malaria season and two during the low malaria season. Individuals in each of three age strata (3–59 months, 5–9 years, and 10 years and above) were sampled in randomly-selected households. Capillary blood was collected by fingerprick, thick and thin blood films were examined. Microscopy was performed at Epicentre, Maradi, Niger, with external quality control. The target sample size was 396 households during the high-season surveys and 266 households during the low-season surveys.

**Results:**

Prevalence of parasitaemia was highest in children aged 5–9 years during all four surveys, ranging between 53.6% (95%CI 48.8–63.6) in February 2018 and 73.2% (66.2–79.2) in September 2017. Prevalence of parasitaemia among children aged 3–59 months ranged between 39.6% (33.2–46.4) in February 2018 and 51.9% (45.1–58.6) in October 2016. Parasite density was highest in children aged 3–59 months during all four surveys, and was higher in high season surveys than in low season surveys among all participants. The prevalence of gametocytaemia in children aged 3–59 months ranged between 9.9% (6.5–14.8) in February 2018 and 19.3% (14.6–25.2) in October 2016. The prevalence of gametocytaemia in children aged 5–9 years ranged between 6.3% (3.5–11.1) in February 2018 and 18.5% (12.7–26.1) in October 2016.

**Conclusions:**

Asymptomatic malaria infection is highly prevalent in this area, even during the season with low incidence of clinical malaria. The high prevalence of parasitaemia in children aged 5–9 years warrants considering their inclusion in SMC programmes in this context.

## Background

Niger is a vast landlocked country in West Africa, with an estimated population of over 21 million [1]. Malaria is the country’s single most important morbidity [1], estimated to have caused over 7.7 million cases and 17,000 deaths in 2017 [2]. Most of the population of the country lives in Sahelian regions of the south of the country, where rainfall is highly seasonal. Because of this, malaria transmission is also highly seasonal, with most cases reported between July and October [3]. The Magaria District in the Zinder Region borders Nigeria and had an estimated population of 674,457 in 2016. The District reported a total of 106,004 cases of malaria in 2018.

Because of the highly seasonal nature of malaria transmission, seasonal malaria chemoprevention was introduced in Niger in 2013, and at the time of this study in 2016–2018 targeted the entire Magaria District (approximately 146,000 children aged 3–59 months in 2018). This strategy is recommended in areas with strong seasonal malaria, and consists of up to four monthly courses of single-dose sulfadoxine-pyrimethamine (SP) and 3 daily doses of amodiaquine (AQ) during the period of highest malaria risk [4]. Meta-analysis of trial data suggests that nearly three-fourths of malaria cases can be avoided [5], and modelling studies suggest that millions of cases and tens of thousands of deaths would be avoided with full-scale roll-out of SMC [6]. When the programme has been implemented at scale, its protective efficacy against malaria has reached 85% [7].

Evidence shows that SMC is well-tolerated and acceptable to the general population in programme settings [8]. Nonetheless, even considering the impressive potential public health benefits of the programme, the remaining burden of disease remains high in many regions of the Sahel, and potential strategies to expand SMC, and to add to SMC, have been investigated. In an area of prolonged seasonal transmission in northern Ghana, malaria incidence was halved in children receiving five rounds of SMC instead of the recommended four rounds [9]. In a stepped-wedge trial in Senegal, expanding the target age groups to include all children under 10 years was shown to reduce the incidence of rapid diagnostic test (RDT)-confirmed malaria in target age groups by 60% [10]. And in Burkina Faso, over a 3-year follow-up period, the combination of seasonal vaccination with RTS,S and SMC was shown to be more effective at preventing malaria and death than either intervention alone [11].

Given the substantial residual burden of malaria in Magaria despite the benefits of SMC, we performed a series of community-based surveys to better understand malaria epidemiology in the Magaria District, and to potentially inform future policy decisions about preventive strategies.

## Methods

### Study design and setting

**Fig. 1:**
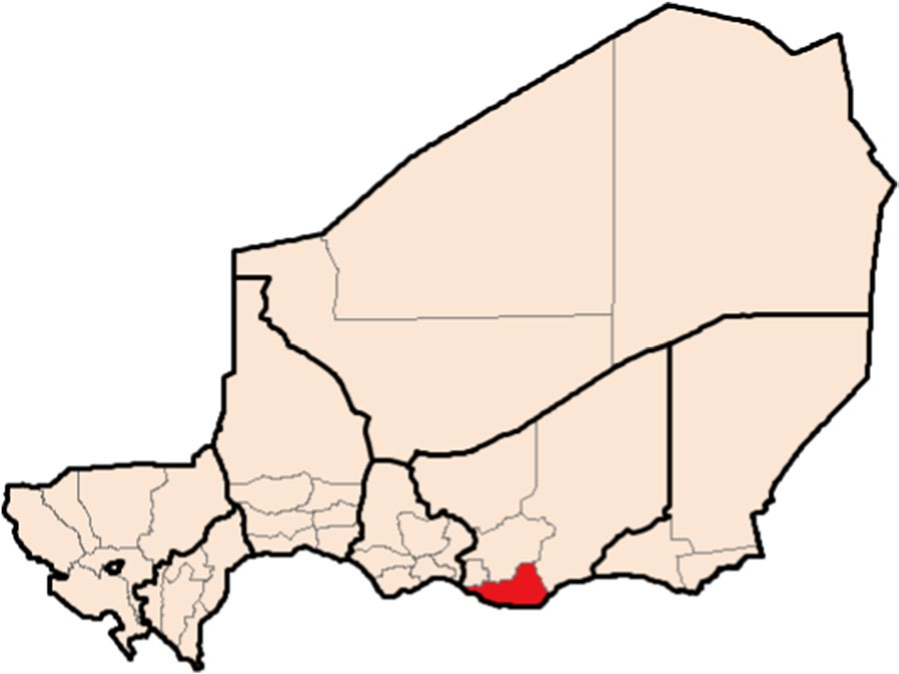
Magaria District in the Zinder Region of Niger

Four cross-sectional, household-based malaria prevalence surveys were performed in the Magaria District of Niger between October 2016 and February 2018 (Fig. 1). Two surveys were performed during the high malaria transmission season (early October 2016 and late September 2017) and two surveys were performed during the low malaria transmission season (late December 2016 and February 2018). Both high season surveys took place in the three days prior to the fourth distribution round of SMC in the study area. The study area was contiguous with the catchment area of health structures serving 140 villages (median village population 473, interquartile range {321,723}, minimum–maximum {68–3350}).

**Fig. 2:**
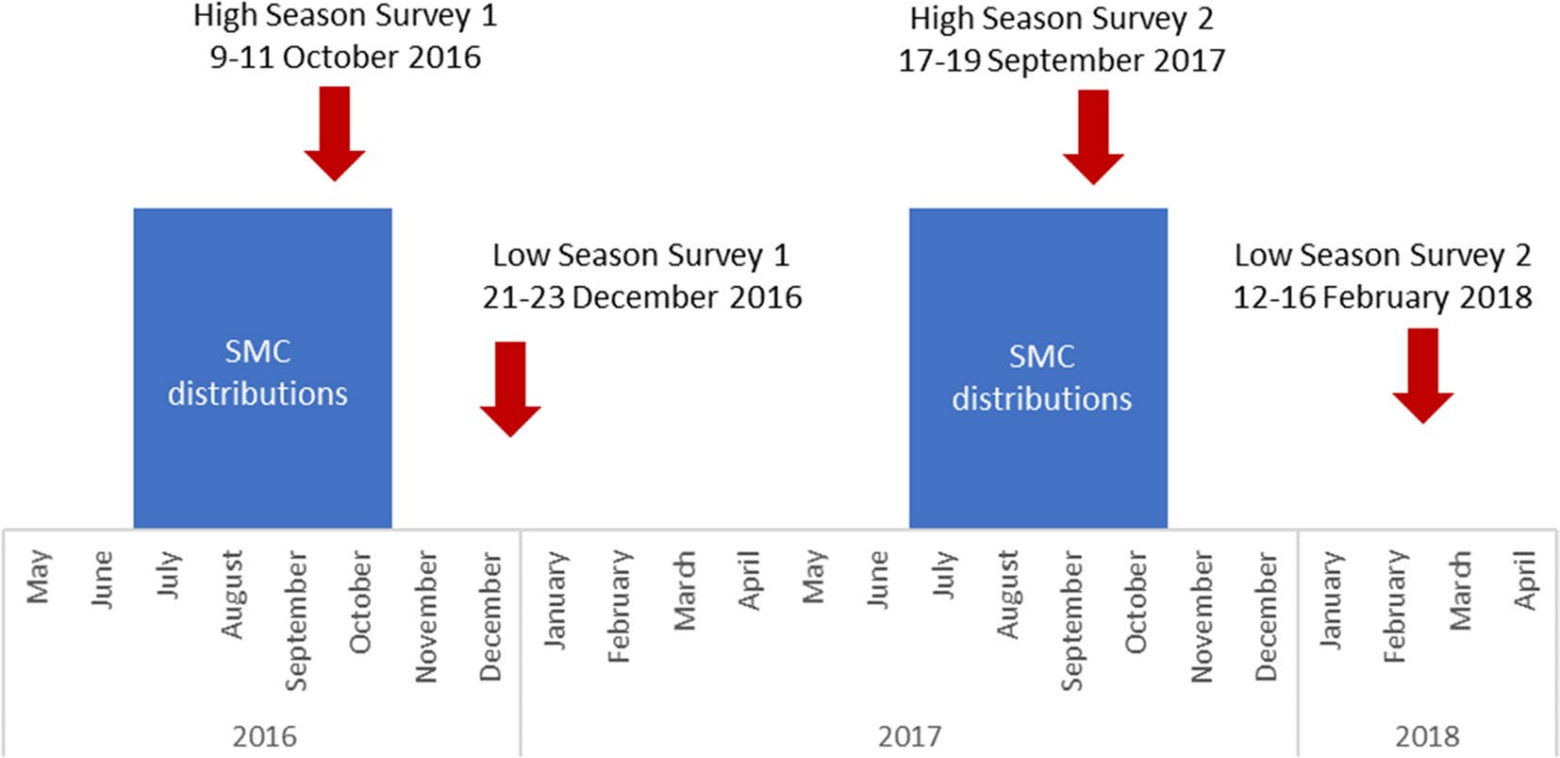
Timing of SMC distributions and malaria prevalence surveys, Magaria, Niger, 2016–2018

During the 2016 season, two distribution strategies were used in the study area. In villages in one contiguous zone, health workers directly-observed the first dose of SMC (SP + AQ1) at a fixed-point distribution site, and the child’s caregiver was given the remaining doses of the blisterpack with instructions for administration at home the following 2 days. This strategy is referred to as the directly observed therapy strategy (DOT). In villages in the second contiguous zone, no doses of SMC were directly observed by health workers at the fixed-point distribution sites, and all doses were administered by caregivers at home. This strategy is referred to as the non-directly observed strategy (non-DOT). During the 2017 season, the DOT strategy was used throughout the study zone. An independent SMC coverage survey performed in 2016 estimated programme coverage of 88% for the first distribution of the season, 90% for the second, 72% for the third and 70% for the fourth. The 2016 high-season prevalence survey described here was performed at the end of the third distribution cycle. No independent SMC coverage surveys were performed in 2017 (see Fig. 2).

### Sampling

In conjunction with district health authorities and local authorities at health structures, a master list of all villages in the study area was created, and a sampling frame including village populations was created in Microsoft Excel. This frame was used to establish the number of households to include in each village, with the probability of inclusion proportional to the size of the population of the village. This process was repeated for each of the 4 surveys.

Individual households were selected using randomly-selected geolocalized coordinates. In each study village, a set of random points was created and overlaid a recent commercially-available satellite image of the village (Google Earth). Those points which fell within 5 m of a building were retained, and numbered sequentially in the village, until the target number of households to include in each village was attained. These points were then loaded into handheld GPS devices and used during the surveys. If the selected building did not contain a household (either because of vacancy or day-long absence), the nearest building to the selected household was visited to replace the missing household. If this second building was also empty, the household was not replaced.

The survey sample consisted of three age-based strata: children aged 3–59 months, children aged 5–9 years, and persons aged 10 years and older. In each household, one person from each stratum was offered enrollment. In a given household, if there were multiple individuals in a given stratum, one person was randomly selected using a random numbers table. All household members (including those who were temporarily absent) were eligible for inclusion. If a temporarily-absent household member was selected, study staff revisited the household later in the day or the following day.

### Sample collection and processing

After obtaining informed consent (and assent from minor participants aged ≥ 10 years), the study nurse collected basic demographic information (age, gender, and village) about the participant on the study register. The participant was also asked whether they slept under a bed net the previous night, and whether they had been febrile in the preceding 24 h.

The study nurse then collected capillary blood by a fingerstick, preparing thick and thin smears on two different slides following study standard operating procedures (SOP). Slides were allowed to dry in a carrier protected from dust and flies and were then transported to the study lab in Magaria at the end of each day.

If a participant or other household member was febrile or reported a history of fever in the preceding 24 h, the study nurse also performed a rapid diagnostic test for malaria (CareStart pLDH(pan), AccessBio, Somerset, NJ, USA). If the RDT was positive and there were no signs of severity, the participant was treated with artemether-lumefantrine according to national guidelines. If a participant or other household member had signs of severe malaria, they were immediately transported to the nearest health centre.

### Laboratory methods

At the end of each day, study staff returned to the central laboratory with the samples collected in the field. Thick and thin smears were stained following study SOPs and were then independently examined by two microscopists following study SOPs and WHO guidance [12]. A minimum of 200 high-powered fields (HPF) were read before reporting a slide as negative. For the quantification of parasitaemia, only asexual forms (trophozoites) were used. In each HPF with between 4 and 20 white blood cells (WBC), the number of trophozoites were counted, until a total of 200 WBCs were seen. If ≤ 9 parasites were seen at that point, the manual counting continued until 500 WBC had been counted. If ≥ 500 parasites were counted before reaching 200 WBCs, the counting stopped at that point, and the formula below was used to quantify parasitaemia.

$$Parasite\,density = \frac{number\,of\,trophozoites\,counted \times 8000}{{number\,of\,WBC\,counted}}$$

In the event of a discordance (different result positive/negative, different *Plasmodium* species identified, or parasite count differing by > 50% between the first and second reads), the laboratory supervisor performed a third examination of the slide. The final reported parasite density was the average of the value reported by the supervisor and the microscopist whose value was closest to that of the supervisor.

External quality control on a random sample of slides read as positive and negative from each survey was performed at the Centre de Recherche Médicale et Sanitaire (CERMES) in Niamey.

### Statistical methods and sample size

The target sample size for each survey was calculated to provide an estimate of the prevalence of microscopy-confirmed parasitaemia in each of the three different age strata, under the assumption that the prevalence would differ among the age groups and between the two surveys.

While it would have theoretically been possible to enroll different numbers of individuals per stratum, this would have been difficult to implement practically. Therefore, for simplicity’s sake, and to reduce the risk of error, to the target was to enroll 360 individuals in each stratum during the high-season surveys (assuming 40% prevalence), and 242 in each stratum during the low-season surveys (assuming 20% prevalence). To account for non-response and households missing members of a given stratum, these figures were increased by 10%. The final target sample sizes were therefore set at 396 households (with one member of each age stratum) in the high transmission season surveys and 266 households in the low transmission season surveys.

For each survey, the prevalence of parasitaemia was independently estimated in each stratum taking into account the survey design, and 95% confidence intervals were calculated. The same procedures were used to describe the prevalence of gametocytaemia in each age group. Participants infected with any *Plasmodium* species were considered to be parasitaemic.

Comparison between groups took into account survey design. Multiple comparisons between groups in a single survey were tested using chi-square, Wald tests and the Kruskall-Wallis tests. Comparisons of the same groups between the four surveys were made using the t-test and the Mann–Whitney test. These comparisons should be regarded as exploratory, as the study was not designed to make these comparisons. Data were analysed using Stata v15.0 (College Station, TX, USA).

## Results

### Description of study participants

393 random points were selected for the first high-season survey. At 129 of the points, both the household located at the random point and its replacement were absent. After inclusion, one household withdrew its consent. A total of 263 households and 591 individuals were therefore included and microscopy results were available for 588 individuals (3 were discarded because of poor-quality slides).

Because of the high absentee rate during the first high-season survey, 300 random points were selected for the first low-season survey. At 32 of the points, both the household located at the random point its replacement were absent. A total of 268 households and 634 individuals were therefore included.

410 random points were selected for the second high-season survey. At 4 points, both the household and its replacement were absent. After inclusion, 3 households withdrew consent. At 30 random points, households refused to participate. A total of 373 households and 818 individuals were therefore included.

266 random points were selected for the second low-season survey. At 6 random points, the household refused to participate. A total of 260 households and 643 individuals were therefore included.

Participants were evenly split by gender, and during each of the surveys, the stratum containing school-aged children had the most missing members from the households (Table 1).

**Table 1:** Description of survey participants, Magaria, Niger, 2016–2018

	High season survey 1 Oct 2016 (N = 588)		Low season survey 1 Dec 2016 (N = 634)		High season survey 2 Sept 2017 (N = 818)		Low season survey 2 Feb 2018 (N = 643)	
	n	%	n	%	n	%	n	%
Gender								
Male	292	49.7	352	55.5	405	49.5	310	48.2
Female	296	50.3	282	44.5	413	50.5	333	51.8
Age								
3–59 months	212	36.1	210	33.1	274	33.5	212	33.0
5–9 years	130	22.1	153	24.1	179	21.9	174	27.1
≥ 10 years	246	41.8	271	42.7	365	44.6	257	40.0
Type of SMC in area								
DOT	327	55.6	300	47.3	818	100	643	100
Non-Dot	261	44.4	334	52.7	–	–	–	–

### Prevalence of parasitaemia and gametocytaemia

The prevalence of parasitaemia is summarized in Table 2. During the first high-season and first low-season surveys, all participants with parasitaemia (254 in high season and 256 in low season) were infected with *Plasmodium falciparum*. During the second high-season survey, of 379 participants with positive blood smears, 361 participants had mono-infection with *P. falciparum*, 10 participants had mixed infection with *P. falciparum* and *Plasmodium ovale*, 2 participants had mixed infection with *P. falciparum* and *Plasmodium malariae*, 5 participants had mono-infection with *P. ovale*, and 1 participant had mono-infection with *P. malariae*. During the second low-season survey, 213 participants had mono-infection with *P. falciparum*, 1 participant had mixed infection with *P. falciparum* and *P. ovale*, 2 participants had mixed infection with *P. falciparum* and *P. malariae*, 2 participants had mono-infection with *P. ovale*, and 2 participants had mono-infection with *P. malariae*.

**Table 2:** Prevalence of parasitaemia and description of parasite density, Magaria, Niger, 2016

	High season survey 1 October 2016 (N = 588)		Low season survey 1 December 2016 (N = 634)		High season survey 2 September 2017 (N = 818)		Low season survey 2 February 2018 (N = 643)	
Gender, % (95% CI)								
Male	42.5	36.7–48.5	41.2	36.1–46.4	47.4	42.5–52.4	36.3	31.4–41.6
Female	43.9	38.0–50.0	39.4	33.8–45.2	45.3	40.7–50.0	31.9	27.0–37.3
Age, % (95% CI)								
3–59 months	51.9^*^	45.1–58.6	48.1^*^	41.4–54.9	50.7^*^	44.8–56.6	39.6^*^	33.2–46.4
5–9 years	66.2	57.5–73.9	64.1	56.1–71.3	73.2	66.2–79.2	56.3	48.8–63.6
≥ 10 years	23.6	18.6–29.4	21.0	16.6–26.2	29.9	25.4–34.8	14.8	10.9–19.7
Type of SMC in the area, % (95%CI)								
Directly-observed first dose	40.1	35.0–45.4	42.7	36.8–48.8	46.3	42.9–49.8	34.2	30.7–37.9
Non-directly-observed first dose	47.1	40.3–54.1	38.3	33.4–43.5	–	–	–	–
Median parasitaemia (parasites/μl), IQR								
3–59 months	6066^†§^	1281–26,065	1320^†^	489–4272	3932^†§^	459–24,312	797^†^	227–1708
5–9 years	1516^§^	530–5705	576	220–1495	1734^§^	495–7380	651	143–1517
≥ 10 years	189^§^	126–629	103	63–159	168^§^	63–676	67	40–173

^*^Adjusted Wald *p* < 0.005 between 3–59 m and 5–9y in all four surveys; *p* < 0.0001 between both groups of children and adults in all four surveys

^†^Kruskall-Wallis *p* < 0.0001 for age in each survey

^§^Mann–Whitney *p* < 0.005 for age groups between high and low-season surveys

Within individual surveys, there were no differences in the prevalence of parasitaemia between males and females. Similarly, there was no difference in parasitaemia between areas receiving different SMC delivery strategies in 2016. While the overall prevalence of parasitaemia did not differ between the first high-season and first low-season surveys conducted in 2016, the prevalence was higher in the second high season survey compared to the second low-season survey, which held across gender and age groups.

In all four surveys, children aged 5–9 years had the highest prevalence of parasitaemia, ranging from a low of 56.3% (95% CI 48.8–63.6) during the second low-season survey to a high of 73.2% (95% CI 66.2–79.2) during the second high-season survey. They were followed by children aged 3–59 months, and then participants ≥ 10 years of age (Table 2). Nonetheless, in all four surveys, among infected individuals, parasite density was highest among children aged 3–59 months, whose parasite densities were significantly higher than those aged 5–9 years. Participants aged ≥ 10 years had the lowest prevalence of parasitaemia in all four surveys, and also the lowest parasite densities.

Regarding gametocytaemia, there were no differences between gender or SMC distribution strategies, but there was a significant trend for decreasing prevalence across the age groups (Table 3). Among children aged 3–59 months, the prevalence of gametocytaemia ranged between a low of 9.9% (95% CI 6.5–14.8) during the second low-season survey and a high of 19.3% (95% CI 14.6–25.2) during the first high-season survey. Despite the overall trend for less gametocytaemia with increasing age, there were no significant pairwise differences in prevalence of gametocytaemia between the youngest participants and those aged 5–9 years during any survey. Prevalence of gametocytaemia among participants aged ≥ 10 years ranged between 1.5% (95% CI 0.6–3.9) during the first low-season survey and a high of 5.5% (95% CI 3.6–8.4) during the second high-season survey but was significantly lower than for younger participants during each survey. Within each age group, the prevalence of gametocytaemia was significantly higher during the high malaria transmission than during the low malaria transmission season.

**Table 3:** Prevalence of gametocytaemia, Magaria, Niger, 2016–2018

	High season survey 1 October 2016 (N = 588)		Low season survey 1 December 2016 (N = 634)		High season survey 2 September 2017 (N = 818)		Low season survey 2 February 2018 (N = 643)	
	Prevalence	95% CI	Prevalence	95% CI	Prevalence	95% CI	Prevalence	95% CI
Gender								
Male	14.4	10.7–19.0	7.4	4.9–11.0	13.3	10.2–17.3	6.0	3.9–9.2
Female	11.8	8.3–17.7	8.5	5.6–12.7	9.7	7.2–13.0	5.2	3.2–8.2
Age								
3–59 months	19.3^*^	14.6–25.2	12.4^*^	8.6–17.6	16.4^*^	12.5–21.3	9.9^*^	6.5–14.8
5–9 years	18.5	12.7–26.1	13.1	8.6–19.4	16.2	11.5–22.4	6.3	3.5–11.1
≥ 10 years	4.9	2.8–8.4	1.5	0.6–3.9	5.5	3.6–8.4	1.6	0.6–4.1
Type of SMC in the area								
DOT	14.1	10.5–18.6	7.7	5.0–11.6	11.5	9.3–14.1	5.6	4.0–7.8
Non-Dot	11.9	8.4–16.6	8.1	5.4–11.9	–	–	–	–

^*^Adjusted Wald *p* < 0.0001 between both groups of children and adults during each survey; no differences between children aged 3–59 months and 5–9 years in any given survey

Results of external quality control were concordant (both in terms of positive/negative result and in terms of parasite density) in 99% of samples sent for testing.

## Discussion

This series of community-based malaria prevalence surveys provides valuable information that adds to our understanding of malaria in an area receiving SMC, and sheds light on new potential strategies for malaria prevention.

Two main findings were expected: that children are more likely to be parasitaemic than adults and that younger children who are infected are more likely to have higher parasite burdens. On the other hand, the fact that children aged 5–9 had a higher prevalence of parasitaemia than children aged 3–59 months in each survey was mildly surprising. Simple explanations would be that older children are less likely to sleep under mosquito nets and have some degree of acquired immunity, but the most obvious explanation for this trend in this area is that the younger children are in the target age group for SMC, while the older group are not. Another explanation would be that children under 5 years old have better overall access to health care because of national policy dictating that their care is free of charge.

Approximately half of children in the community were carrying parasites during the high-season surveys, which occurred just under 4 weeks after the previous SMC distribution. The following distribution might have decreased the overall parasite burden among this group, but the results from the low-season surveys clearly show that even with SMC there is a significant residual parasite burden in this population at the end of the high-transmission season. These findings should help counter fears that SMC may lead to increased rates of severe malaria in older children (by prevention acquisition of natural immunity), because children in this area are clearly exposed to malaria parasites, even with a functional SMC programme.

Malaria transmission is driven by the presence of gametocytes in peripheral blood. The high prevalence of gametocytaemia among children aged 3–59 months during the high season surveys is not particularly surprising, but the fact that there was no difference in the prevalence of gametocytaemia between children aged 3–59 months and children aged 5–9 years shows that this older population is significantly contributing to malaria transmission in the community. These school-aged children have likely developed significant natural immunity to malaria after repeated infections in early childhood, hence their lower parasite densities.

These surveys were undertaken to better understand the epidemiology of malaria in this area with extremely strong and seasonal transmission. A case–control study performed in the same study zone suggested that SMC was generally effective at preventing clinical malaria, despite poor adherence to AQ at home [13]. Despite its effectiveness at preventing clinical illness in this area, there is still a considerable parasite burden among the SMC target population. It should nonetheless be noted that the two high-season surveys took place at the end of an SMC cycle, when the prophylactic effect of the medicines is expected to be lowest.

The coverage of SMC in the study area was reasonably high, though it could certainly have been improved. Efforts should be made to consolidate coverage in the current target group of children. Nonetheless, given the results of these surveys, it is reasonable to question whether SMC is targeted at the optimal group of children in rural Niger. A survey in another part of rural Niger showed similar results, with asymptomatic *Plasmodium* carriage high in school-aged children [3]. These children aged 5–9 years contribute significantly to the overall population parasite burden, have the highest proportion of parasitaemia, and are some of the major drivers of transmission. These children have likely been repeatedly exposed to *Plasmodium* infection over time and are, therefore, likely not at high risk of developing severe malaria, but given the high prevalence of parasitaemia, expanding the target age group of SMC to children aged 5–9 years might prevent significant morbidity in this population.

The largest potential benefit of an SMC expansion would likely be indirect. With a high prevalence of gametocytaemia, children aged 5–9 are helping to drive transmission in their communities, in turn causing malaria infections (more likely to be severe) among their younger siblings and neighbours. Evidence supporting this hypothesis has already been shown in a stepped-wedge trial in Senegal, where the expansion of SMC to children under 10 was associated with a 27% reduction in malaria in persons who had not received SMC, an effect that was not seen when SMC was limited to children 3–59 months [10].

SMC was never intended to be a magic bullet, there are many logistical challenges inherent with implementing SMC year in and year out in the context of the rural Sahel[14]. Doubling the target population would not be easy, as it would require additional financial and logistical resources. School-aged children could be reached by school-based strategies, which might be easier to implement and lead to greater treatment adherence. On the other hand, concerns about the drug pressure exerted by SMC on antimalarial resistance patterns would be amplified if more children were targeted. Given the added costs (even if some implementations costs might be mitigated by economies of scale with existing programme, any expansion of SMC would also need to be accompanied by appropriate evaluations – including measures of effectiveness (both direct and indirect) and, if possible, cost-effectiveness. The choice about how best to invest limited resources – expanding the target or consolidating coverage in the current target group – is not simple.

There are limits to these surveys. The most important is that the target sample sizes were not met for all strata in the surveys. In the first survey, the absentee rate was much higher than expected. This was overcome with more intensive preparation in subsequent surveys, but many households with adult respondents did not have children in one of the two age groups. In some cases, this was because of older or younger heads of household without children present, and in others it was due to the absence of school-aged children. This has led to larger confidence intervals than expected, particularly for the children aged 5–9 years, but it is reassuring that the overall trends are consistent between the surveys. Information about receipt of (or adherence to) SMC in the children in the youngest age group was not collected, though it has been previously shown that adherence in the study area was poor in 2016 [13]. The timing of the two low-season surveys was not identical, so comparisons should be guarded. Another limitation is the classification of the ages of children, which despite the use of event calendars, can sometimes be approximate when precise birthdates are unknown. It is, therefore, possible that some participants, particularly those around the threshold of 5 years, may have been misclassified.

## Conclusions

These surveys showed a high prevalence of asymptomatic parasitaemia among children under 10 years old in an area of rural Niger where children under 5 were receiving SMC. The high prevalence of parasitaemia in school-aged children could justify expansion of SMC to this group, but this decision would need to consider clinical disease as well as financial and logistic concerns.

## Data Availability

The datasets generated and analysed during this study are available from the corresponding author on reasonable request.

## Abbreviations

AQ: Amodiaquine
DOT: Directly-observed therapy
GPS: Global positioning system
HPF: High-powered field
RDT: Rapid diagnostic test
SMC: Seasonal malaria chemoprevention
SP: Sulfdoxine-pyrimethamine
WBC: White blood cell
WHO: World Health Organization
